# “Psyosphere”: A GPS Data-Analysing Tool for the Behavioural Sciences

**DOI:** 10.3389/fpsyg.2021.538529

**Published:** 2021-05-13

**Authors:** Benjamin Ziepert, Peter W. de Vries, Elze Ufkes

**Affiliations:** Department of Psychology of Conflict, Risk, and Safety, University of Twente, Enschede, Netherlands

**Keywords:** GPS, positioning technologies, implicit measurement, spatial movement, walking, psychology

## Abstract

Positioning technologies, such as GPS are widespread in society but are used only sparingly in behavioural science research, e.g., because processing positioning technology data can be cumbersome. The current work attempts to unlock positioning technology potential for behavioural science studies by developing and testing a research tool to analyse GPS tracks. This tool—psyosphere—is published as open-source software, and aims to extract behaviours from GPSs data that are more germane to behavioural research. Two field experiments were conducted to test application of the research tool. During these experiments, participants played a smuggling game, thereby either smuggling tokens representing illicit material past border guards or not. Results suggested that participants varied widely in variables, such as course and speed variability and distance from team members in response to the presence of border guards. Subsequent analyses showed that some of these GPS-derived behavioural variables could be linked to self-reported mental states, such as fear. Although more work needs to be done, the current study demonstrates that psyosphere may enable researchers to conduct behavioural experiments with positioning technology, outside of a laboratory setting.

## Introduction

Positioning technologies, such as Global Positioning Systems (GPS), Glonass, and Galileo can be used to determine the position on the globe and to record, for instance, the movement of planes, cars, and individuals (Hofmann-Wellenhof et al., 2007). Positioning technologies are now omnipresent in mobile devices, such as smart phones, tablets, and laptops, which makes them potentially interesting for the study of behaviour in naturalistic settings. It could, for instance, be used to identify people with early warnings signs for depression (Saeb et al., 2015; Palmius et al., 2017), partly or fully replace self-reported diaries in traffic research (Stopher et al., 2002; Wolf, 2006; Bohte and Maat, 2009; Schuessler and Axhausen, 2009), determine how populations behave after a disaster, such as an earthquake (Bengtsson et al., 2011), or to automatically detect active pickpockets in a shopping mall (Bouma et al., 2014). Surprisingly, behavioural scientists have so far used positioning technologies only sparingly.

Arguably, there are two reasons why positioning technologies have largely been neglected in behavioural research. First, the data are too complex to analyse with software that are traditionally used in the social sciences, such as IBM SPSS Statistics (SPSS). Second, only a limited number of studies has so far investigated the relationship between psychological variables and positioning technology data. Consequently, little information is available about which psychological variables could be studied with positioning technologies.

Therefore, the aim of this study is to develop and test a tool that enables social scientists to conduct behavioural experiments with positioning technology. The tool will be tested with a “proof of principle” study, to illustrate that aspects of people’s movements could be used to shed light on their states, and the study will explore possible relationships rather than testing specific hypotheses.

In current behavioural science research, the assessment of movement is often done via trained observers, interviewers, or self-reported diaries. Although these methods have been of great use (Goodchild and Janelle, 1984; Janelle et al., 1988; Doherty and Miller, 2000; Axhausen et al., 2002; Shoval et al., 2010), they do have their drawbacks. Shoval et al. (2010), for instance, point to the bias in participants’ self-reported movements. For example, people frequently underreport small trips and trips that do not start or end at home. Moreover, participants driving a car tend to underestimate travel time, whereas public transportation users often overestimate it (Stopher, 1992; Ettema et al., 1996). Furthermore, participants may decide to omit information, such as when they view the information as socially undesirable. Finally, interviewers could fail to prompt recall (interviewer error), or the participants could simply forget the information over time (recall bias; Anderson, 1971; Golledge, 1997; Vazquez-Prokopec et al., 2009). These limitations can be circumvented by using positioning technologies, such as GPS (Bohte and Maat, 2009).

Especially in traffic research, positioning technologies, such as GPS have been argued to have several benefits over traditional methods of movement tracking (Stopher et al., 2002; Wolf et al., 2004; Bohte and Maat, 2009; Schuessler and Axhausen, 2009). Compared to self-reported diaries or interviews, for instance, GPS loggers are less effortful to work with, as they may substantially reduce information that otherwise needs to be self-reported by participants or needs to be asked by interviewers. As a result, GPS loggers may draw less heavily on interviewers and may therefore reduce the costs of conducting studies. In addition, they afford longer survey periods, as smartphone apps tracking movement in the background allow for longer data-collection periods compared to when the participants self-report their trips. Moreover, use of GPS may increase the quality of the data gathered, as travel times are recorded accurately regardless of the length of the data-collection period, whether they include one’s home or not, and whether they are spent driving or walking. Finally, the sensors also have the benefit of recording additional data, such as speed and acceleration which can be used for additional analysis (Wolf et al., 2004).

Studies in other fields have also employed positioning technologies to replace or augment traditional methods of movement tracking, especially research with target groups that are unable to maintain a self-reported diary, such as the mentally impaired, children, and the elderly (Shoval et al., 2011). Measuring movement in such groups typically requires enlisting the help of caretakers or family members, registering their activities or filling in behavioural checklists in their stead (Shoval et al., 2011). This often turns out to be relatively expensive, burdensome and biased, even to the point where researchers may avoid conducting studies with these target groups altogether (Isaacson et al., 2016). In such contexts, replacing human observers with GPS loggers may prove useful (Shoval et al., 2008, 2010, 2011; Isaacson et al., 2016), and the required protocols can be followed by mentally impaired and elderly people (Isaacson et al., 2016).

Table 1 shows that whereas positioning technologies have been taken up by quite a few researchers, studies specifically investigating the link between positioning-technology data and psychological variables, especially mental states, are few and far between. This is surprising, given that several laboratory studies have already managed to establish such links with more conventional means. For instance, sad, depressed and frightened people tend to walk slower than others; additionally, joy and anger are linked to increased walking speed (Michalak et al., 2009; Barliya et al., 2012; Gross et al., 2012). Other research indicates that personality traits, such as agreeableness are also linked to increased walking speed (Satchell et al., 2017). Similarly, positive affect, extraversion, or openness to experiences have been associated with physical activity (Byrne and Byrne, 1993; Schwerdtfeger et al., 2010). Insofar GPS has been used in behavioural research, it has focused on constructing measures of daily activity (Wolf et al., 2013; Fillekes et al., 2019; also see Carlson et al., 2014; Jankowska et al., 2015; James et al., 2016), to detect risk-taking behaviour on the road, i.e., speeding (Bolderdijk et al., 2011), or to predict depression and social anxiety (Saeb et al., 2015; Huang et al., 2016; Palmius et al., 2017). However, links between location-based data and state variables have, so far, received little attention, if any at all.

**TABLE 1:** Positioning technologies and their use in past research.

Measures	Research
Anxiety, depression, or lifestyle (e.g., positive affect or extraversion)	Determining relationship between active vs. sedentary lifestyle, social anxiety and depression, and number places visited with GPS (Wolf et al., 2013; Huang et al., 2016; Saeb et al., 2016).
Community specific routes description and visualisation	Measuring segregation in city communities with GPS (Davies et al., 2017; Whyatt et al., 2017).
Depression detection	Detecting depression from GPS movement data characteristics, such as location variance, home stay, or mobility between favourite locations (Saeb et al., 2015; Palmius et al., 2017).
Environmental exposure	Measuring daily environmental exposure with GPS (Phillips et al., 2001; Chaix et al., 2013).
Following and leadership detection	Detecting leadership and followership with movement patterns (e.g., co-moving) with Wi-Fi data (Kjargaard et al., 2013).
Information or disease spreading characteristics	Tracking the spreading of information in face-to-face networks with Bluetooth, RFID, and Wi-Fi (Madan et al., 2010; Isella et al.,2011a,b).
Physical activity and mobility	Measuring physical activity of children, the elderly, or other target groups with GPS (Elgethun et al., 2002; Fjørtoft et al., 2009; Maddison and Mhurchu, 2009; Krenn et al., 2011; Shoval et al., 2011; Carlson et al., 2014; Isaacson et al., 2015; Jankowska et al., 2015; James et al., 2016; Fillekes et al., 2019).
Pickpocket detection	Detecting pickpockets with movement characteristics (e.g., walking speed) measured with security cameras (Bouma et al., 2014).
Population movement characteristics	Identify population behaviour after a disaster with GSM (Bengtsson et al., 2011).
Risk seeking	Measuring speeding as a form of risk seeking with GPS (Bolderdijk et al., 2011).
Travel characteristics, such as travel mode, route choice, or speed	Studying travel behaviour, such as travel mode choice, route choice or speed with GPS (Murakami and Wagner, 1999; Draijer et al., 2000; Wolf, 2000, 2006; Stopher et al., 2002; Wolf et al., 2004; Bohte and Maat, 2009; Schuessler and Axhausen, 2009; Necula, 2015).
Virus transmission risk	Determining the spreading of disease with GPS (Vazquez-Prokopec et al., 2009, 2013).
Walking routes	Assessing tourist walking routes with GSM and GPS (Xia et al., 2008).

Arguably, the apparent lack of interest among behavioural scientists, especially psychologists, can be explained by a limited awareness that positioning technologies may yield information that sheds light on psychological state variables. We therefore decided to develop and test a tool that extracts different aspects of behaviour from GPS logs, i.e., to enable behavioural scientists to analyse movement data without the need of additional special expertise. This tool was subsequently applied to field-experimental data to see whether these behavioural aspects can be linked to various state variables.

Out of the variety of positioning technologies we decided to focus on GPS for our tool. GPS can be used all over the globe and does not depend on local GSM, Wi-Fi, or other infrastructure. GPS is also omnipresent in smart phones or other devices, and dedicated GPS loggers are affordable. The data analysis software will work with longitude, latitude, and timestamp data points that are typical for GPS loggers. The movement data from other positioning technologies, such as GSM and Wi-Fi data can be converted to be used with the same software once it is converted to longitude and latitude.

The tool is an R package called “psyosphere” (Ziepert et al., 2018; see Supplementary Appendix 1). R is an open-source programming language and data-analysis tool that is becoming more widespread (Muenchen, 2012). The choice for R has several benefits: since R is used by psychologists and computer scientists it could improve cooperation of interdisciplinary teams, the software is free of charge and therefore more easily accessible than, for instance, SPSS, there are pre-existing packages for spherical calculations and handling of GPS data (e.g., Kahle and Wickham, 2013; Hijmans et al., 2015; Loecher and Ropkins, 2015; Wickham, 2016), and since R is open-source software psyosphere can be improved upon by the research community. Furthermore, psyosphere allows researchers to analyse movement between persons (e.g., by calculating distances between persons while they are moving on different paths) and within-person differences (e.g., measuring movement changes over time). Additionally, it is possible to add custom measures to psyosphere.

Research outside of the laboratory has shown that pickpocketing corresponds with specific body movements (Bouma et al., 2014). Their algorithms to detect pickpockets were based on variations in walking speed, orientation change or distance to other people, and were shown to have a sensitivity up to 95.6% with 0.5% false alarms. Similarly, the mental processes while smuggling, i.e., transporting an illegal package, may also lead to changes in behaviour that could be detected by the observers (Wijn et al., 2017).

The mental processes involved when transporting illegal substances can be linked to hostile intent. Wijn et al. (2017) define hostile intent “as an individual’s intent to act in ways that imply or aim to inflict harm onto others” (p. 2), and although with smuggling the harm inflicted on others is perhaps more indirect and systemic—i.e., harm to the end users’ health and society—they share essential characteristics. Indeed, people with hostile intent try to hide it when they expect that others will try to prevent their actions (Ekman et al., 1988; DePaulo et al., 2003; Koller et al., 2016; Wijn et al., 2017), and this should be no different for those who carry contraband. Furthermore, Wijn et al. (2017) suggest that the risk of being detected and apprehended—which would apply to smugglers as well—predisposes the psychological mind set of those harbouring such intentions towards increased anxiousness, self-focus, and vigilance. This, in turn, may influence their responses to environmental cues, specifically those that signal risk of exposure, causing a fear-related response pattern (e.g., fight, flight, or freeze) while transporting illegal substances. To illustrate how risk of exposure can influence body movement, a study on lie detection showed that participants became more rigid in their movement when instructed to tell a lie. Moreover, even when the participants were told that moving less would give them away, participants were unable to correct for the increased rigidity (Vrij et al., 1996; Van der Zee et al., 2019).

The current study focuses on establishing links between state variables and movement-descriptive variables in a smuggling game, requiring participants to transport legal or supposedly illegal material across a border area at the risk of being apprehended by border guards. As there is little in the way of theory to guide selection and construction of movement-specific variables in this context, the selection of behavioural variables—speed, speed variation, distance to peers, distance from a shortest route, and variation in the distance from the shortest route—was based on somewhat liberal interpretation of whatever literature was available.

The first variable, Speed, has been linked in past research to mental states and traits (Michalak et al., 2009; Bolderdijk et al., 2011; Barliya et al., 2012; Gross et al., 2012; Satchell et al., 2017), has been used successfully in detecting pickpockets (Bouma et al., 2014), and has also emerged as a strategy to avoid fear- and stress-inducing stimuli (Krieglmeyer et al., 2013). Moreover, environmental factors can influence walking speed. For instance, walking in nature decreases walking speed compared to urban settings, and walking speed decreased more when the setting was more natural (e.g., higher trees; Franěk and Režný, 2017). Interestingly, this effect could be linked to an environmental stressor, i.e., traffic noise (Franěk et al., 2018); apparently, stress caused by exposure to noise motivated people to minimise exposure to it by walking faster. Other work also shows that stimulus evaluations evoke approach-avoidance behaviours, e.g., suggesting that negatively evaluated stimuli cause people to try and increase the distance between them and the stimulus (Krieglmeyer et al., 2013). Thus, smugglers fearing apprehension by border guards could for instance be expected to walk faster.

Distance to peers (Intra-Team Distance) is the mean distance from one person to the other persons within a group and past research has shown that people stay closer together when confronted with an outside threat (Schachter, 1959; Feshbach and Feshbach, 1963; Brady and Walker, 1978) and additionally, people keep a larger interpersonal distance when they are in uncertain situations with a threat to personal self-esteem (Schachter, 1959; Brady and Walker, 1978). Consequently, when transporting illegal substances and encountering a police officer, people could, for instance, be expected to walk closer together when they are afraid or walk further apart when they believe they are doing something illegal.

The variable Speed Variation is calculated as the standard deviation of the Speed between a start and finish of a series of coordinates. The distance from a shortest route (Route Deviation) is calculated as the distance between the measured points and the shortest possible route between two locations, and the variation in the distance from the shortest route (Variation Route Deviation) is the standard deviation of the Route Deviation for all data points between two locations. Subsequently, Route Deviation would increase if a person moves away further from the shortest route and Variation Route Deviation would increase if a person meanders while moving between two locations. To our knowledge, all three measures have not been used in past research. However, they could be argued to tie in either with fear-related response patterns, such as fight, flight, and freeze responses (Wijn et al., 2017), and rigidity (Vrij et al., 1996), or with strategies to avoid becoming close to a fear- or stress-inducing stimulus, i.e., the border guard (e.g., Krieglmeyer et al., 2013). Thus, a person transporting an illegal substance and encountering a guard may try to act normal by continuing on a straight path and keeping the same pace (cf. Vrij et al., 1996; Wijn et al., 2017), while avoidance tendencies could lead to behaviour changes, such as walking away from the guard, taking a longer route, changing the route more often or changing pace.

The proposed measures could also be used in other situations to study fear-related response patterns in combination with marked events. For instance, fear and avoidance responses can be measured when approaching meetings, classes, outgroup members or work environments, and may be used to analyse anxiety, depression, group association or workplace morale. Specifically, Intra-Team Distance, could be used to assess interpersonal relationships, fear, or feelings of guilt at a workplace environment. Moreover, Route Deviation and Variation Route Deviation could be used in wayfinding studies to assess if people are certain about their route taken, and Speed Variation could be used to analyse movement patterns during mass events, for instance, stop-and-go movement patterns signalling overcrowding in a specific area (Moussaïd et al., 2011).

To exemplify the possible use of psyosphere and the proposed measures, the current study encompasses two field experiments conducted as part of an undergraduate course for psychology students. During the experiments, the participants would wear GPS loggers and would transport cards either representing legal or supposedly illegal material across a border area with guards that could apprehend them and confiscate the cards. After each round, various state variables were measured. The two experiments serve as a “proof of principle,” that given a certain context (i.e., hostile intent), aspects of people’s movement could relate to their mental states. Thus, the experiments explore possible relationships rather than testing specific hypotheses pertaining to the behaviour and mental states of smugglers vs. non-smugglers.

## Materials and Methods

### Participants

Two similar experiments were conducted as part of an undergraduate psychology course at the University of Twente. The first experiment took place in 2014 and the second in 2015. In the first experiment 69 students participated, who all received a GPS logger. Two were excluded from the analysis due to failure of GPS loggers and five others served as guards, and so 62 students (44 female and 18 male) remained as participants. The average age was 21.61 (*SD* = 5.60) and ranged from 18 to 37. Furthermore, 30 students were Dutch and 32 were German.

In the second experiment 91 students participated; the 80 available GPS loggers were randomly distributed among them. Of the 80 participants with loggers, three served as guards, and another three suffered from sensor failure. The remaining 74 students (51 female and 23 male) had an average age of 22.41 (*SD* = 5.60), ranging from 18 to 46; 38 were Dutch and 34 were German. The participants that acted as guards (five in Exp. 1, three in Exp. 2) were excluded from analysis to limit the scope of the current study.

Power analyses were not conducted in order to determine a required number of participants; instead, we were constrained by the number of students who had enrolled in the courses in 2014 and 2015, respectively, as well as the numbers of GPS loggers that were available. For logistical and practical reasons, additional data collection under comparable circumstances in order to meet pre-determined numbers of participants was virtually impossible.

### Procedure

The participants signed up for the experiment during an introductory lecture for an undergraduate course. The experiment was explained to the participants and they received written instructions. Afterwards they signed an informed consent form. On the basis of randomness participants were either assigned to one of the smuggling teams or to act as one of the guards. In Exp. 1 group sizes ranged between three and six (*M* = 4.77, *SD* = 0.83); in Exp. 2 they ranged between two and six (*M* = 4.63, *SD* = 1.36). The participants received a GPS logger and were told to gather 3 h later in a small park on the university campus.

#### Tasks

The teams (smugglers) had the task to transport supposed legal and illegal material and the other participants, i.e., guards, were required to intercept those with illegal material. This “material” consisted of a card the size of a playing card that either displayed cocaine (“illegal card”) or flour (“legal card”). They received both legal and illegal cards in the starting area, and distributed these among each other, making sure that each participant carried one (either legal or illegal). The cards stated that the teams would win 10 points for each illegal card they transported and one point for each legal card. Guards would also score 10 points for intercepting in illegal card, but lose one point if they intercepted a legal card. The best team’s members and best guard would each win a cinema voucher and a chocolate bar.

Before starting the teams had to write their GPS logger’s number and the starting time on the card.

#### Area

**FIGURE 1:**
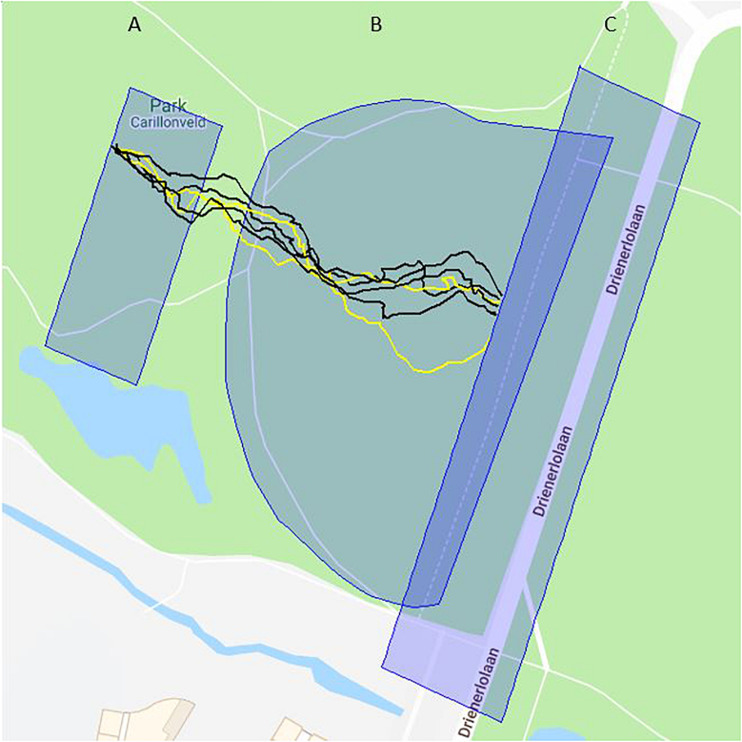
Experiment area with participant tracks and GPS polygons. The tracks of six team members in Experiment 2 are plotted in black (illegal card) and yellow (legal card). They started in polygon **(A)**, crossed the semi-circular bicycle path into the area where they would be visible to the guards (polygon **B**), and ended before the finish (polygon **C**).

After arriving at the park, participants were directed to their assigned locations. The teams would go to a starting point that was behind a mount and out of sight of the guards. The guards were waiting at the finish in an approximately 2-m-wide and 20-m-long strip that the teams were required to cross. The finish area was marked with barrier tape on the ground. A group of 17 tall trees were standing inside and around the finish area. A visual inspection of the data did not reveal signal distortions by the trees. The distance from the starting area to the finish area was 150 m. Roughly halfway between start and finish was a semi-circular bicycle path that was slightly elevated; as a result, the teams and guards could observe each other only after the former had walked onto the bicycle path. The guards were positioned in the centre of the semi-circle in about 75 m from the edge of the semi-circle. See Figure 1 for an illustration of the area.

#### Round

The teams were instructed to walk from the starting area, across the park and through the finish area. The guards could confiscate the team members’ card by tapping them on their shoulder. The team member would give their card to the guard and the guard would note a number on the card which was assigned to the guard. The team members had to avoid being checked by the guards. This could be done by, for instance, distracting them by sending the team members with legal cards first or walking with a wide distance among each other therefore it would be difficult for the guards to reach all team members before they crossed the finish area. The guards were not allowed to leave the finish area and had to wear safety vests to enable the team members to spot them easily. Each time after crossing the finish area the team members would drop the remaining cards they had into a box and fill in a questionnaire. After this they would walk back to the start position for another Round.

#### Experiment 1

Five participants were assigned to be guards and the other participants formed teams of three to six members, with an average of 4.77. Before the start of the first experiment the participants filled in an additional trait questionnaire. Further, participants were instructed not to run, and four rounds were conducted. Additionally, all teams were wearing a card with their team number on their chest. One team of five participants was asked to wear skin conductivity sensors to measure stress levels, but these all failed. At the starting area each team received a set of cards, two of which were “illegal” cards; teams could decide themselves who would smuggle. Afterwards, between four and five teams with an average of 21.08 participants would start at the same time and the ratio between guard’s to participants was 0.24 when the participants approached the finish area. After each Round the teams would fill in a Dutch version of the State questionnaire (see Supplementary Appendix 2).

#### Experiment 2

Experiment 2 differed slightly from 1. First, three (rather than five) participants were made guards; the others formed two- to six-member teams, with an average of 4.63. Furthermore, the instruction not to run was omitted, and the participants were not wearing any stress sensors or team numbers. At the starting area the teams could freely choose the ratio of illegal and legal cards and they were asked to write down which strategy they wanted to use. Additionally, the finish area in Experiment 2 was larger than in Experiment 1 and enabled the guards to walk more freely. At the end of each Round, the team members would write down their points and could see the total points of the other teams. Finally, they would only fill in an English version of the State questionnaire. Some questions were removed and a few were added in the State questionnaire; for instance, participants were asked to rank the extent to which they thought each team member acted as a leader, and to describe how they thought they could improve their strategy as a team in the next round (these are beyond the scope of this paper, however). The guards were frequently informed how many points they had obtained.

### Measures

#### State Questionnaire

The mental states of the participants were measured with a questionnaire based on Stekkinger (2012) and Wijn et al. (2017). Some questions were amended for a better fit to the current context and two questions were added to measure self-observed behaviour changes (e.g., whether the participants changed their pace after seeing the guards). Supplementary Appendix 2 contains all questions, moreover, Supplementary Appendix Tables 10, 11 report the Cronbach’s alpha or Pearson’s *R* for the state questionnaire constructs. The reliabilities of all scales were above 0.78. All State questions used a 7-point Likert scale from 1 “Not at all” to 7 “Very much.”

Two items checked to what extent participants experienced hostile intentions (Hostile Intent; Stekkinger, 2012; Wijn et al., 2017). Five items measured the participants alertness to threats from the guards (Alertness to Being Target of Guards; Galbraith et al., 2008; Stekkinger, 2012; Wijn et al., 2017). Five items checked the inhibitory and activation control (Cognitive Self-Regulation; Stekkinger, 2012; Wijn et al., 2017). Four items measured the self-focus of the participants (Situational Self Awareness; Govern and Marsch, 2001; Stekkinger, 2012; Wijn et al., 2017). Four items assessed the feelings of fright that the participants felt through the presence of the guards (Frightened by Presence of Guards; Stekkinger, 2012; Wijn et al., 2017). Five items checked the impulses that were suppressed by the participants (Suppressed Impulses to Change Movement; Stekkinger, 2012). Three items measured the extent that participants questioned themselves (Contemplation of Hostile Intent; Stekkinger, 2012; Wijn et al., 2017). Finally, two items were added to the questionnaire that assessed the self-observed behaviour changes of participants (Awareness Movement Change in Presence of Guards; Stekkinger, 2012). For a detailed explanation of the mental processes and their function, see Wijn et al. (2017).

#### GPS Data

Every participant carried an i-gotU GT-600 GPS logger with a SiRF Star III GPS Chip and a L1, 1575.42 MHz frequency. The sensors were turned on at least 5 min before the start of the experiments and checked if they were recording. Every second, the logger received time, latitude, longitude, and elevation and saved these in a data point. From the GPS data Speed, Speed Variation, Intra-Team Distance, Route Deviation and Variation Route Deviation were calculated in 1-s increments using the R package psyosphere. Speed was measured as the mean kilometres per hour between each data point. Speed Variation was calculated as the standard deviation of the kilometres per hour between each data point between start and finish. Intra-Team Distance is the mean distance from one team member to the other team members in metres. Route Deviation is the distance in metres between the data points and the shortest route from start to finish. Variation Route Deviation is the standard deviation of the Route Deviation for all data points from start to finish. The start was the point where a participant entered the starting area (see polygon A in Figure 1) and the finish was the point where a participant crossed the finish line (see polygon C in Figure 1). Therefore, the exact start and finish point could differ for each participant.

### Analysis

#### GPS Data Preparation

The data from the GPS loggers were exported and analysed with the R package psyosphere, developed for the current study (Ziepert et al., 2018). The software created a track for each Round of each participant, and plotted the tracks on a map retrieved from Google maps (Google LLC, 2018). Polygons could be defined to mark areas of specific interest (see Figure 1 for map, tracks, and polygons).

A track began in the starting area that was determined by a polygon of GPS coordinates (polygon A) and ended when the participants crossed a GPS based finish line behind the finish area (polygon C). In between was the area where team members and guards could actually see each other (polygon B). Before the point of visibility, the teams generally followed a straight line and started to change their movement after seeing the guards. Therefore, the analysis included only the data from the point where teams became visible to the guards and vice versa, to where the members crossed the finish line. Within this line-of-sight area 31,113 coordinates were recorded in Experiment 1, for four rounds, and 17,172 in Experiment 2, for three rounds. These data were used by the R package to calculate the GPS variables mentioned above.

The R package detects two types of faulty data. First, an unrealistic speed can, for instance, be recorded due to temporary signal loss from the GPS satellites. Therefore, if the speed exceeded 40 km/h the data was excluded from analysis. This occurred 16 times (0.05%) in Experiment 1 and 8 times (0.05%) in Experiment 2. Second, if the time difference between coordinates exceeded 1 s then the Speed, Speed Variation and Distance between the coordinates were marked as missing values and also excluded from analysis. Three coordinates (0.01%) in Experiment 1 and 152 coordinates (0.89%) in Experiment 2 were excluded because of a time difference larger than one second. Additionally, two sensors in Experiment 1 and three sensors in Experiment 2 stopped recording all GPS satellite data and had to be excluded from the analysis.

#### State PCA

We analysed the State questions that were used in both experiments with a principal component analysis (PCA) and a confirmatory factor analysis (CFA). In total, six explorative PCAs were conducted, one for each of the three rounds in the two experiments. Afterwards, we compared the PCAs and counted how often items shared a component. A model with eight components emerged and was subsequently tested with a CFA.

#### Relationships Between State and GPS Variables

Descriptive statistics and correlations, for the State components and GPS variables, were calculated for each experiment separately. Finally, a multi-level analysis was conducted with the GPS variables as dependent variables, and the State components and rounds as the predictors. In total, 10 models were created, 5 for each experiment. The multi-level analysis tested for consistent changes per Round (e.g., increasing Intra-Team Distance per Round) and the impact of grouping in teams. Three random effect models did not converge, and two of these models were models with a maximum random-effects structure based on the experimental design. Moreover, according to Barr et al. (2013), a maximum random-effects model should be prioritised when conducting a multilevel analysis. Therefore, we choose for a maximum random-effects model with random slopes per Round and a static intercept per team and participant. To improve the model convergence rate, Barr et al. (2013) suggest to remove outliers, and therefore, data have been removed from the GPS variables, except Intra-Team Distance, when the data were outside of the Inter Quartile Range times 1.5 (Hoaglin, 2003). Fifteen outliers have been removed from Speed, nine from Speed Variation, five from Route Deviation, and fourteen from Variation Route Deviation. After removing the outliers, all models with a maximum random-effects structure converged. Intra-Team Distance was excluded from the outlier removal since this increased the model convergence rate.

#### Exclusions

Only the first three rounds of both experiments were used since the participants did not complete the State questionnaire after the fourth Round of Experiment 1. For educational purposes, a limited number of participants in Exp. 1 were fitted with skin conductivity to measure stress levels; these data could not be used due to sensor malfunctions. Questions pertaining to intended strategy changes in the next round, leadership and motivation were also outside of the scope of this paper and are not analysed. In Exp. 1 two GPS loggers failed, and in Exp. 2 three.

## Results

We first conducted principal component analyses (PCA), confirmatory factory analyses (CFA), and invariance model comparisons for 30 items of the state questionnaire for each experiment and for each of the first three rounds. The questionnaire can be found in Supplementary Appendix 2. The descriptive statistics, correlations tables, and the CFA results can be found in Supplementary Appendix 3; a section describing notable differences between the experiments can also be found in this Supplementary Appendix.

### Regression Analysis

Per experiment and for each of the five GPS-based outcome variables regression analyses were conducted with the state constructs as predictors. These 10 regression models were tested for random effects per participant, team, and Round. Testing for random effects is necessary since the measurements for each participant are not independent but depend on the Round that is measured and the team a participant is in. For instance, a team with fast walking members could have motivated a slow walking member to walk faster. Barr et al. (2013) suggest to select a maximum random-effects model based on the experimental design instead of selecting a model based on the model fit. For our current study, a maximum random-effects model includes random slopes per round and a static intercept per team and participant (see Eq. 1). Finally, we were interested in the effect of all state variables on the GPS outcome variable (e.g., speed), and consequently, the State variables were added as predictors to the regression model. See Eq. 2 for a simplified version of the fixed effects formula.

(1) $$∼R\,o\,u\,n\,d|\frac{T\,e\,a\,m}{P\,a\,r\,t\,i\,c\,i\,p\,a\,n\,t}$$

G⁢P⁢S=I⁢l⁢l⁢e⁢g⁢a⁢l⁢C⁢a⁢r⁢d⁢S⁢e⁢l⁢e⁢c⁢t⁢i⁢o⁢n⁢β+S⁢e⁢l⁢f⁢a⁢s⁢T⁢a⁢r⁢g⁢e⁢t⁢β+A⁢w⁢a⁢r⁢e⁢n⁢e⁢s⁢s⁢C⁢o⁢g⁢n⁢i⁢t⁢i⁢v⁢e⁢B⁢e⁢h⁢a⁢v⁢i⁢o⁢u⁢r⁢C⁢h⁢a⁢n⁢g⁢e⁢β

(2) +S⁢i⁢t⁢u⁢a⁢t⁢i⁢o⁢n⁢a⁢l⁢S⁢e⁢l⁢f⁢A⁢w⁢a⁢r⁢e⁢n⁢e⁢s⁢s⁢β+F⁢r⁢i⁢g⁢h⁢t⁢β+I⁢m⁢p⁢u⁢l⁢s⁢e⁢β+D⁢u⁢b⁢i⁢o⁢u⁢s⁢T⁢h⁢o⁢u⁢g⁢h⁢t⁢s⁢β+A⁢w⁢a⁢r⁢e⁢n⁢e⁢s⁢s⁢P⁢h⁢y⁢s⁢i⁢c⁢a⁢l⁢B⁢e⁢h⁢a⁢v⁢i⁢o⁢u⁢r⁢C⁢h⁢a⁢n⁢g⁢e⁢β

#### Model for Speed

We calculated Model 6.2 with Speed as the outcome variable and Table 2 displays the results per estimate. As Table 2 highlights, Awareness Movement Change in Presence of Guards was a significant and positive predictor for Speed in Experiment 1 and the same relationship was not significant in Experiment 2 (*b*_1_ = 0.08, *p*_1_ < 0.001, *b*_2_ = 0.05, *p*_2_ = 0.241). Thus, the more participants reported a speed or route change after seeing the guards, the faster they walked. Likely, they did so in an attempt to outpace or evade the guards.

**TABLE 2:** Regression beta, SE, and *P*-values for speed as dependent variable.

	Experiment 1			Experiment 2		
Estimate	*b*	*SE*	*p*	*b*	*SE*	*p*
Round	0.05	0.04	0.232	0.11	0.07	0.116
Illegal Card Selection	–0.03	0.07	0.636	0.04	0.18	0.819
Alertness to Being Target of Guards	–0.03	0.02	0.166	–0.01	0.04	0.721
Cognitive Self-Regulation	–0.02	0.02	0.395	0.01	0.05	0.832
Situational Self Awareness	–0.01	0.03	0.687	–0.05	0.05	0.339
Frightened by Presence of Guards	0.03	0.03	0.290	–0.00	0.05	0.951
Suppressed Impulses to Change Movement	**–0.09**	0.03	**0.008**	–0.07	0.05	0.195
Contemplation of Hostile Intent	0.02	0.03	0.527	–0.03	0.05	0.537
Awareness Movement Change in Presence of Guards	**0.08**	0.02	**<0.001**	0.05	0.04	0.241

**P-values less than 0.050 are in bold.**

Additionally, in Experiment 1 Suppressed Impulses to Change Movement is a significant and negative predictor for Speed though the same relationship was not significant in Experiment 2 (*b*_1_ = –0.09, *p*_1_ = 0.008, *b*_2_ = –0.07, *p*_2_ = 0.195). This means that people who suppressed their impulses also walked slower, and an explanation could be that participants walked slower in order not to attract the attention of the guards. An alternative explanation could be that participants were uncertain which route would be the best to avoid the guards and therefore slowed their pace.

#### Model for Speed Variation

We calculated Model 6.2 with Speed Variation as the outcome variable and Table 3 displays the results per estimate. The table shows that, Suppressed Impulses to Change Movement was a significant a positive predictor in Experiment 1 but not in Experiment 2 (*b*_1_ = 0.12, *p*_1_ < 0.001, *b*_2_ = 0.03, *p*_2_ = 0.575). This means that when the participants had suppressed impulses, then they varied their walking pace more. A simple explanation could be that participants failed in suppressing their impulses and therefore varied more. However, as Table 2 shows, participants reduced their pace when they had suppressed impulses (*b*_1_ = –0.09, *p*_1_ = 0.008, *b*_2_ = –0.07, *p*_2_ = 0.195) and if participants had failed in suppressing their impulses, one would suspect that their overall pace would have increased and not decreased. Hence, an alternative explanation could be that Suppressed Impulses to Change Movement measures the uncertainty of the participants on how to avoid the guards rather than suppressed impulses. Accordingly, the uncertainty could have caused the participants to slowdown, in order to orient themselves, and then to follow the new path with an increased pace.

**TABLE 3:** Regression beta, SE, and *P*-values for speed variation as dependent variable.

	Experiment 1			Experiment 2		
Estimate	*b*	*SE*	*p*	*b*	*SE*	*p*
Round	**0.08**	0.04	**0.031**	0.05	0.09	0.575
Illegal Card Selection	0.11	0.06	0.088	–0.16	0.22	0.449
Alertness to Being Target of Guards	**0.05**	0.02	**0.008**	0.07	0.04	0.139
Cognitive Self-Regulation	–0.03	0.02	0.217	0.07	0.06	0.285
Situational Self Awareness	0.04	0.02	0.117	–0.11	0.06	0.069
Frightened by Presence of Guards	–0.03	0.03	0.207	–0.01	0.06	0.885
Suppressed Impulses to Change Movement	**0.12**	0.03	**<0.001**	0.03	0.06	0.575
Contemplation of Hostile Intent	–0.05	0.03	0.065	–0.04	0.06	0.468
Awareness Movement Change in Presence of Guards	**-0.06**	0.02	**0.002**	0.00	0.05	0.933

**P-values less than 0.050 are in bold.**

Furthermore, Round is a positive and significant predictor for Speed Variation in Experiment 1; the same relationship is not significant in Experiment 2, however (*b*_1_ = 0.08, *p*_1_ < 0.031, *b*_2_ = 0.05, *p*_2_ = 0.575). Consequently, with each consecutive Round the participants varied more in their pace, and the variation could have helped the participants to avoid the guards better.

Additionally, Alertness to Being Target of Guards is a positive and significant predictor for Speed Variation, though the same relationship was not significant in Experiment 2 (*b*_1_ = 0.05, *p*_1_ = 0.008, *b*_2_ = 0.07, *p*_2_ = 0.139). Namely, participants feeling targeted by the guards varied their speed more. A likely explanation is that participants tried to avoid the guards by changing their walking pace.

Finally, Awareness of Movement Change in Presence of Guards was a significant and negative predictor for Speed Variation in Experiment 1 but not in Experiment 2 (*b*_1_ = –0.06, *p*_1_ = 0.002, *b*_2_ = 0.00, *p*_2_ = 0.993). This means that participants’ awareness that they changed their route or speed after seeing the guards, corresponded with less variation in their walking pace. A reason could be, that participants had chosen a route after seeing the guards that successfully avoided the guards and therefore the participants could keep their pace. Because of the lower guard ratio in Experiment 1, it was easier to avoid the guards than in Experiment 2.

#### Model for Intra-Team Distance

Model 6.2 was calculated with Intra-Team Distance as the outcome variable and Table 4 displays the results per estimate. The table shows that Round was a positive and significant predictor for Intra-Team Distance in Experiment 1 and the same relationship was close to significant in Experiment 2 (*b*_1_ = 2.24, *p*_1_ < 0.003, *b*_2_ = 0.85, *p*_2_ = 0.067). This means that the distance to other team members increased with each Round, for instance, implying the emergence of a strategy to better avoid the guards.

**TABLE 4:** Regression beta, SE, and *P*-values for intra-team distance as dependent variable.

	Experiment 1			Experiment 2		
Estimate	*b*	*SE*	*p*	*b*	*SE*	*p*
Round	**2.24**	0.73	**0.003**	0.85	0.46	0.067
Illegal Card Selection	0.17	0.58	0.770	0.08	0.70	0.906
Alertness to Being Target of Guards	0.18	0.18	0.323	0.16	0.14	0.262
Cognitive Self-Regulation	0.13	0.19	0.497	0.27	0.18	0.129
Situational Self Awareness	–0.32	0.20	0.119	–0.06	0.18	0.719
Frightened by Presence of Guards	**–0.62**	0.27	**0.023**	–0.20	0.17	0.242
Suppressed Impulses to Change Movement	0.19	0.27	0.479	–0.01	0.18	0.953
Contemplation of Hostile Intent	**0.52**	0.24	**0.034**	0.09	0.15	0.580
Awareness Movement Change in Presence of Guards	–0.07	0.16	0.665	–0.13	0.15	0.413

**P-values less than 0.050 are in bold.**

Furthermore, Frightened by Presence of Guards was a significant and negative predictor for Intra-Team Distance in Experiment 1 while the same relationship was not significant in Experiment 2 (*b*_1_ = –0.62, *p*_1_ = 0.023, *b*_2_ = –0.20, *p*_2_ = 0.242). Therefore, when participants had feelings of fright because of the guards then they walked closer together, possibly to compensate for their fear. Alternatively, observing that they walked close together may also have made them realise they were more likely to be a target, which may in turn have inspired feelings of fright.

Additionally, Contemplation of Hostile Intent was a significant and positive predictor for Intra-Team Distance in Experiment 1 and the same relationship was not significant in Experiment 2 (*b*_1_ = 0.52, *p*_1_ = 0.034, *b*_2_ = 0.09, *p*_2_ = 0.580). The more participants were questioning the legality of their actions or whether they have to hide something, the further they would walk apart from their fellow team members. It is possible, the participants had conflicting emotions about their hostile intentions and therefore did not want to affiliate with their team members.

#### Model for Route Deviation

Model 6.2 was conducted with Route Deviation as the outcome variable and Table 5 displays the results per estimate. As the table shows, Alertness to Being Target of Guards is a significant and positive predictor for Route Deviation in Experiment 2 and a close to significant and negative predictor in Experiment 1 (*b*_1_ = –0.32, *p*_1_ = 0.068, *b*_2_ = 0.62, *p*_2_ = 0.031). Therefore, in Experiment 1, participants that perceived themselves as a target by the guards kept a shorter distance to the shortest route, and in Experiment 2, participants did the opposite. The difference could be explained by the change in the guard ratio. In Experiment 1, the guard had to be selective and participants who felt they were a target could try to act as normal as possible by deviating less from the shortest route. In Experiment 2, the guards could stop all participants if they were fast enough, and therefore, when participants would feel themselves a target would need to actively avoid the guards by outwalking them.

**TABLE 5:** Regression beta, SE, and *P*-values for route deviation as dependent variable.

	Experiment 1			Experiment 2		
Estimate	*b*	*SE*	*p*	*b*	*SE*	*p*
Round	**2.14**	0.52	**<0.001**	0.18	0.53	0.738
Illegal Card Selection	–0.26	0.58	0.656	–1.89	1.32	0.155
Alertness to Being Target of Guards	–0.32	0.17	0.068	**0.62**	0.28	**0.031**
Cognitive Self-Regulation	–0.26	0.21	0.226	0.42	0.39	0.279
Situational Self Awareness	0.11	0.22	0.624	–0.10	0.37	0.778
Frightened by Presence of Guards	–0.27	0.28	0.335	–0.06	0.36	0.875
Suppressed Impulses to Change Movement	**0.63**	0.28	**0.028**	0.28	0.39	0.484
Contemplation of Hostile Intent	0.01	0.26	0.958	–0.52	0.35	0.138
Awareness Movement Change in Presence of Guards	–0.04	0.18	0.825	0.25	0.31	0.415

**P-values less than 0.050 are in bold.**

Additionally, Round is a significant and positive predictor for Route Deviation in Experiment 1, but the same relationship is not significant in Experiment 2 (*b*_1_ = 2.14, *p*_1_ < 0.001, *b*_2_ = 0.18, *p*_2_ = 0.738). This means that each Round participants walked further away from the shortest route. Possibly, they increasingly realised that a higher route deviation helped in avoiding the guards. Additionally, the higher guard ratio in Experiment 2 made it easier to pursue participants and therefore Route Deviation was greater in Experiment 2 from the start than in Experiment 1, and consequently, could not be increased as much as in Experiment 1.

Finally, Suppressed Impulses to Change Movement is a significant and positive predictor for Route Deviation in Experiment 1 and the same relationship was not significant in Experiment 2 (*b*_1_ = 0.63, *p*_1_ = 0.028, *b*_2_ = 0.28, *p*_2_ = 0.484). Therefore, participants who reported suppressed impulses also deviated more from the shortest route. An explanation could be that participants were uncertain about their route and therefore deviated more from the shortest route.

#### Model for Variation Route Deviation

We calculated Model 6.2 with Variation Route Deviation as the outcome variable and Table 6 displays the results per estimate. As the table shows, Suppressed Impulses to Change Movement is a significant predictor for Variation Route Deviation in Experiment 1 and Experiment 2 (*b*_1_ = 0.34, *p*_1_ = 0.033, *b*_2_ = 0.37, *p*_2_ = 0.017). The more participants reported suppressed feelings, the more often they changed their routes. A reason could be that the participants were uncertain about the route to avoid the guards and therefore changed it more often.

**TABLE 6:** Regression beta, SE, and *P*-values for variation route deviation as dependent variable.

	Experiment 1			Experiment 2		
Estimate	*b*	*SE*	*p*	*b*	*SE*	*p*
Round	**0.82**	0.26	**0.002**	0.08	0.22	0.734
Illegal Card Selection	0.10	0.33	0.773	**-1.05**	0.53	**0.049**
Alertness to Being Target of Guards	–0.14	0.10	0.164	**0.29**	0.11	**0.009**
Cognitive Self-Regulation	–0.19	0.12	0.118	0.24	0.15	0.119
Situational Self Awareness	0.12	0.13	0.368	–0.19	0.15	0.188
Frightened by Presence of Guards	–0.20	0.16	0.207	–0.21	0.14	0.150
Suppressed Impulses to Change Movement	**0.34**	0.16	**0.033**	**0.37**	0.15	**0.017**
Contemplation of Hostile Intent	0.01	0.15	0.944	–0.23	0.14	0.093
Awareness Movement Change in Presence of Guards	**0.23**	0.10	**0.024**	0.08	0.12	0.529

**P-values less than 0.050 are in bold.**

Additionally, Round is a negative and significant predictor for Variation Route Deviation in Experiment 2 and the opposite relationship is not significant in Experiment 1 (*b*_1_ = 0.10, *p*_1_ = 0.773, *b*_2_ = –1.05, *p*_2_ = 0.049). Thus, those who selected an illegal card in Experiment 2 changed their route less often. An explanation is the small number of participants that carried a legal card in Experiment 2 and which were used by the teams to distract the guards by changing their route more often.

Furthermore, Illegal Card Selection is a negative and significant predictor for Variation Route Deviation in Experiment 2 but is not significant in Experiment 2 (*b*_1_ = 0.12, *p*_1_ = 0.721, *b*_2_ = –1.10, *p*_2_ = 0.045). Therefore, when participants chose an illegal card in Experiment 2 they changed their route less often. An explanation could be that participants with a legal card changed their route more often to attract the attention of the guards and that participant with an illegal card did the opposite. This strategy could have been more important in Experiment 2 since the area where the guards were allowed to walk was larger in Experiment 2. Consequently, when a participant got the attention of the guard, the guard had to walk further away from other participants.

Moreover, Alertness to Being Target of Guards is a positive and significant predictor for Variation Route Deviation in Experiment 2 but is not significant in Experiment 1 (*b*_1_ = –0.14, *p*_1_ = 0.164, *b*_2_ = 0.29, *p*_2_ = 0.009). This means that the more participants perceived themselves as a target by the guards, the more participants would change their route in Experiment 2 while the opposite happened in Experiment 1. The reason could be that in Experiment 2 every participant could be checked and when the participants perceived themselves as a target they actively avoided the guards by changing their route more often. In Experiment 1, in contrast, not all participants could be checked and the participants could try to act normally to reduce guard suspicion.

Finally, Awareness Movement Change in Presence of Guards is a positive and significant predictor for Variation Route Deviation in Experiment 1, although the same relationship is not significant in Experiment 2 (*b*_1_ = 0.23, *p*_1_ = 0.024, *b*_2_ = 0.08, *p*_2_ = 0.529). Thus, participants’ awareness that they had changed their route or speed after seeing the guards corresponded with actual behaviour. A reason could be that the participants attempted to outmanoeuvre the guards by changing the route after seeing them.

### Summary

In summary, the results show that the participants used strategies to avoid the guards. For instance, the participants changed their behaviour with each consecutive Round, by increasing the distance to team members, by accelerating and decelerating more often, by taking longer routes, and by changing the route more often. These changes may indicate a collective strategy by the participants to become better in avoiding the guards. Additionally, according to their descriptions in Experiment 2, teams made use of a distraction strategy: they chose to carry a legal card and distracted the guards, so as to improve the chances of their illegal-card carrying team members.

Participants were presumably uncertain about the best route to avoid the guards, and this may have become overt in more changes in direction, reduced pace, more changes in pace, and increased route lengths. Additionally, participants stayed closer to team members when they had feelings of fear and kept a greater distance if they had the feeling that they had to hide something. Furthermore, participants attempted to avoid guards by changing the pace more often when targeted, by increasing the pace after seeing the guards, and by changing the route more often after seeing the guards.

In Experiment 1 the participants had two illegal cards per team and in Experiment 2 the participants could choose a free ratio of legal and illegal cards. Therefore, the increased availability of illegal cards presumably reduced the relationship between the selection of an illegal card and feeling of hostile intent. Another difference between the experiments was the ratio of guards and participants. Specifically, in Experiment 2 were more guards per participant than in Experiment 1 and that made it more difficult for the participants to avoid the guards in Experiment 2. Consequently, when participants perceived themselves as target by the guards, the participants in Experiment 1 took a more direct path and made less changes to their direction in order not to attract further attention by the guards. Moreover, the participants in Experiment 2 did the opposite, in an attempt, to outmanoeuvre the guards and took a longer route and made more changes to their route. Finally, in Experiment 1, when participants saw the guards they reduced their speed in order not to attract any attention and a similar effect could not be found in Experiment 2.

## Discussion

The aim of the current study was to develop a research tool that enables behavioural scientists to more easily use positioning technologies, such as GPS, for psychological experiments. Additionally, two experiments were conducted as cases against which to test this new research tool, psyosphere.

### Psyosphere

The R package psyosphere (Ziepert et al., 2018) analyses GPS data by transforming GPS tracks into descriptive variables, such as speed, direction or distance, that can be analysed with linear regression methods. It is optimised to handle multiple tracks simultaneously and to make comparisons between these tracks. This is done because comparisons between multiple participants with linear regression methods is a typical technique of conducting studies in behavioural science. To give a simplified example, the speed of multiple car drivers for a given route could be compared to investigate if speed warnings reduce risky driving behaviour. Furthermore, the package supports data preparation through cleaning up the data by marking coordinates with unrealistic speeds as missing values or by detecting measuring gaps. Additionally, sub-tracks can be selected by providing start and finish areas. The package also supports the visualisation of tracks. For this purpose, tracks and polygons can be plotted on maps, tracks can be coloured based on grouping variables, and tracks can be plotted per participant or groups of participants (e.g., teams; see Figure 1).

Psyosphere builds on existing R packages (e.g., Kahle and Wickham, 2013; Hijmans et al., 2015; Loecher and Ropkins, 2015; Wickham, 2016) and on recent work also aiming to unlock the potential of location-based data for psychologists (Geyer et al., 2019). Whereas, Geyer et al. (2019) predominantly focused on creating an Android app allowing accurate location logging and secure storage of data, and, in addition, offer assistance with subsequent data analysis, the psyosphere package focuses specifically on the latter. It complements this earlier work by increasing the possibilities for analyses and enhancing the potential of location data even further. By breaking up a sequence of timestamped coordinates into fine-grained facets of movement behaviours, psyosphere may be of interest for behavioural and psychological researchers.

### Psychological Variables

To illustrate which type of variables could be studied with positioning technologies and psyosphere, an overview of variables that were used in past research was provided (see Table 1). Additionally, as a test case, we conducted two experiments to analyse the relationship between several psychological variables (i.e., feelings of hostile intent and several related states) and movement data measured with GPS loggers. The two experiments have shown relationships that have face validity, correspond with literature, or occur in both experiments. Due to the differences in experimental procedures, both studies cannot be seen as direct, exact replications, which may have caused some findings to occur in only one.

For instance, one finding was that participants who reported higher levels of fear also tended to walk closer together. This finding is in line with past research, demonstrating that people stay closer together when confronted with an outside threat (Schachter, 1959; Feshbach and Feshbach, 1963; Brady and Walker, 1978). In light of the correlational nature of our research, however, this finding is amenable to other interpretations as well.

Additionally, when participants were contemplating whether they were doing something illegal and whether they had to hide something, this corresponded with larger distances to their team members. Congruently, participants in uncertain situations with a threat to personal self-esteem have been shown to keep a larger interpersonal distance (Schachter, 1959; Brady and Walker, 1978). Although self-esteem was not measured here, the item to what extent participants believed that they were doing something illegal could be interpreted as self-esteem related.

Furthermore, participants developed evasive strategies over the three rounds to avoid the guards. In detail, the participants spread out more, took longer routes and changed their route and pace more often. Presumably, the evasive strategies gave the guards fewer opportunities to stop participants and check whether they had illegal cards. Similarly, the second experiment suggested that teams used distraction strategies to improve the overall team score. To distract the guards, one or two team members would carry a legal card, would walk ahead of the team members, with illegal cards, and would show an erratic movement, such as changing the route more often to attract the attention of the guards. In a somewhat similar pen-and-paper experiment, researchers asked participants to draw a route from a starting position to a designated target, without giving away their final destination (Jian et al., 2006). The experiments showed that participants would take a longer route with erratic movement, such as changing direction more often, to hide their intended target. The findings of Jian et al. (2006) are comparable with the evasion strategies observed in the current study and we argue that participants will use evasive strategies if they judge this to be normal, i.e., when other people around them also perform this behaviour as well. Furthermore, participants can use evasive movements that deviate from they believe to be normal movements to purposefully create suspicion.

Finally, when participants were presumably uncertain about their route, they also showed erratic movement, such as changing the route more often, taking longer routes, changing the pace more often and overall walking slower. To test whether participants were actually uncertain, a future study could include related measures. An alternative explanation could be that participants felt regret about the route they chose because they got caught. It would therefore be beneficial to include regret-related measures in future studies as well.

### Limitations

Arguably, the ratio between participants and guards differed between the two studies, and this may have influenced the relationship between the self-reported mental states and the measured GPS variables. Specifically, when the participants were carrying illegal cards, we assumed that they would try to hide this fact before the guards and would try to act normal. To act normal, the participants had to suppress fear-related responses, such as running away. Furthermore, the suppression of fear-related responses requires effort, and cues from the surroundings, such as encountering a guard or being targeted by a guard, could limit the ability of participants to act normal. Therefore, we measured whether the participants changed their movement when they encountered the guards. In the second experiment it was much more likely that participants would be stopped than in the first experiment. As a consequence, the guards were much less selective in stopping participants in the second experiment, and therefore, participants opted more for openly evading guards than trying to act normal. Thus, the higher guards-to-participants ratio may have been the reason that a smaller number of significant relationships between mental states and GPS variables was found in the second experiment compared to the first experiment. Nevertheless, we believe to have found some meaningful relationships, and we advise that future research should limit the number of guards to ensure that not all participants can be checked.

Another limitation of the current study is that we tested for 90 regression estimates, which renders the probability of finding statistically significant relationships merely by chance (Type I error inflation) rather high. In principle, it is possible to correct for this chance by reducing the significance level with, for instance, a Bonferroni correction (Holm, 1979), but it is a matter of long-standing methodological discussion exactly when and how this significance level should be adjusted (e.g., Fisher, 1956; Cabin and Mitchell, 2000). However, as we set out to merely explore our field-experimental data using the psyosphere tool to assess whether it can indeed be used to supplement more conventional means of data gathering, rather than testing specific hypotheses pertaining to the behaviour and mental states of smugglers vs. non-smugglers, we feel it is justified to simply accept a higher risk of false positives (for a discussion, see Wigboldus and Dotsch, 2016). For the current study we therefore chose not to correct the significance level, and, instead, focused on those findings that have some face validity, correspond with similar findings elsewhere, or are sufficiently robust to occur in both experiments.

The explorative nature of this exercise unfortunately sheds little light on the ecological validity of the findings presented here. Indeed, without evidence on the contrary, it would be safest to assume that interpretation of observed behaviour varies between contexts. Walking closer together, for instance, was associated with fear, but in other contexts, such as shopping areas or festivals, might be more likely as a sign of enjoyment in the company of friends. Similarly, changing route and pace was interpreted here as a deliberate strategy to avoid a guard, but in a way-finding context might just as well mean that one does not know where to go, of cannot make up one’s mind about which path to choose.

The objective of this exercise, however, was not to find universal behavioural correlates of mental states per se, but to yield “proof of principle,” i.e., that, given a certain context, aspects of people’s movements could be used to shed light on their states. In this sense, the analyses provide here appear to have succeeded—be it in part. The possibility that these behavioural aspects may imply different emotions, cognitions, or intentions in different contexts is by no means detrimental to our conclusion. However, it does imply that researchers should exercise caution in interpreting such correlations. Extensive pilot testing of which aspects of observed behaviour are correlated with self-reported measures under controlled circumstances before going out into the field is key.

### Future Research and Developments

On a more technical note, past research has shown that detecting movement patterns is dependent on the sensor accuracy (Kjargaard et al., 2013). An older version of the sensors that were used in the current study had an accuracy between 2.50 and 20 m (Vazquez-Prokopec et al., 2009). Research has shown that sensor accuracy can be greatly improved by combining multiple satellite systems, such as GPS, Glonass, Galileo, and BeiDou. These accuracy improvements will allow detection of movement patterns in more detail, making it easier to link them to cognitive processes.

Alternatively, also Wi-Fi and GSM signals can be used to determine the location. Smart phones, internet-of-things (IOT) devices, and specialised hardware can record the Wi-Fi and GSM signals and deduce locations (Kjargaard et al., 2013). It is also possible to track Wi-Fi and GSM-enabled devices from a GSM tower (Bengtsson et al., 2011) or with Wi-Fi routers (Sevtsuk, 2009) even if the devices are not connected to the network. Cameras (Burgess et al., 2014), Bluetooth (Madan et al., 2010), and RFID (Isella et al., 2011a) are yet other technologies offering data that, after conversion to (X, Y) coordinates are amenable to psyosphere analyses.

Thus, psyosphere may facilitate research in a number of domains, such as crowd control during events and behaviour of people in emergencies. Sime (1995), for instance, argued for the importance of psychological processes and phenomena during emergency evacuations. In two-thirds of the time needed to evacuate people in an emergency psychological processes play a pivotal role; only one third of this time is determined by parameters of a strictly technological nature. Sime (1995) argued that it takes time for people to discontinue their ongoing activities, and to seek contact with social others to reduce uncertainty. Whereas these findings were based on interviews with survivors of disasters a considerable time after they happened, collection of location data during emergency drills or actual emergencies—i.e., in real life—and subsequent analyses with psyosphere may well help understand what happens when people need to vacate buildings. This better understanding can, in turn, inform more effective evacuation strategies.

Similarly, psyosphere may facilitate understanding of crowd dynamics. Location data extracted from camera footage or based on signals emanating from smart phones present in crowds may yield insights on crowd members’ moods or the cohesion of groups within the crowd, as well as the way in which these change as a result of policing strategies or attempts at communication by organisers.

Finally, practitioners and scientists working in the field of environmental design may use the tool to study the influences of, for instance, sign-posting or building layout on way-finding; relatively subtle variations in behaviour may point to states as decisiveness, goal-directedness, and confusion. Similarly, the effect of nudges, for instance implemented in nightlife areas to reduce related noise and public urination (e.g., Bloeme et al., 2017), on visitors’ behaviours and their states may be studied in more detail. Added into the bargain, using location data in such settings could eventually absolve researchers from parsing countless hours of video footage.

Once again, however, any such attempt at interpreting behaviour of individuals and groups requires tightly controlled experiments dedicated to establishing causal relationships between aspects of behaviour and state variables.

Furthermore, future research could extend the use of psyosphere by adding features, such as Kjargaard et al.’s (2013) time-lag method for detecting leadership and followership, or apply more complex methods, such as machine-learning classification. The data from studies, such as the current one might, for instance, be used to train an algorithm to establish links between aspects of movement or other behaviours and various psychological state and trait variables, such as having depression (Wolf et al., 2013; Huang et al., 2016; Saeb et al., 2016), or being a pickpocket (Bouma et al., 2014).

## Conclusion

Hopefully, the findings presented here will encourage social scientists to use positioning technologies to study movement outside of a laboratory and in a real-world setting. Moreover, they show that psyosphere can prepare GPS data from psychological experiments for analysis with commonplace statistical methods, such as linear regression.

## Data Availability Statement

The datasets generated for this study are available on request to the corresponding author.

## Ethics Statement

The studies involving human participants were reviewed and approved by the Behavioural, Management, and Social Sciences Ethics Committee of the University of Twente. The participants provided their written informed consent to participate in this study.

## Author Contributions

BZ, PV, and EU jointly conceived the research. BZ and PV collected the data. BZ conducted the analyses and drafted the manuscript. All authors are responsible for substantial revisions of the manuscript, and read and approved this submission.

## Conflict of Interest

The authors declare that the research was conducted in the absence of any commercial or financial relationships that could be construed as a potential conflict of interest.
