# Supplementary material for: “Psyosphere”: A GPS Data-Analysing Tool for the Behavioural Sciences
Source: Front Psychol. 2021 May 13;12:538529. doi: 10.3389/fpsyg.2021.538529 (PMC8155254; doi:10.3389/fpsyg.2021.538529)
Supplement: Supplementary file 1 [file Data_Sheet_1.docx]

## Appendix 1

## Description of “psyosphere” on CRAN

“psyosphere” is published on the Comprehensive R Archive Network (CRAN). The description of “psyosphere” on CRAN is as following: “Analyse location data such as latitude, longitude, and elevation. Based on spherical trigonometry, variables such as speed, bearing, and distances can be calculated from moment to moment, depending on the sampling frequency of the equipment used, and independent of scale. Additionally, the package can plot tracks, coordinates, and shapes on maps, and sub-tracks can be selected with point-in-polygon or other techniques. The package is optimized to support behavioural science experiments with multiple tracks. It can detect and clean up errors in the data and resulting data can be exported to be analysed in statistical software or geographic information systems (GIS).” (Ziepert et al., 2018).
