# Supplementary material for: “Psyosphere”: A GPS Data-Analysing Tool for the Behavioural Sciences
Source: Front Psychol. 2021 May 13;12:538529. doi: 10.3389/fpsyg.2021.538529 (PMC8155254; doi:10.3389/fpsyg.2021.538529)
Supplement: Supplementary file 2 [file Data_Sheet_2.docx]

## Appendix 2

State questionnaire. For all questions a 7-point Likert scale was used ranging from 1 “Not at all” to 7 “Very much”. The questionnaire for the first experiment was in Dutch and for the second in English.

State questionnaire

| Construct | Question | Experiment |
| --- | --- | --- |
| Alertness to Being Target of Guards 1 | I had the feeling the border guard(s) targeted me | 1 and 2 |
| Alertness to Being Target of Guards 2 | I thought I had attracted the border guards’ attention | 1 and 2 |
| Alertness to Being Target of Guards 3 | I had a feeling that I was going to be stopped | 1 and 2 |
| Alertness to Being Target of Guards 4 | I felt like I was the one being addressed by the border guard(s) | 1 and 2 |
| Alertness to Being Target of Guards 5 | I had the idea that the others were paying attention to me | 1 and 2 |
| Frightened by Presence of Guards 1 | I was startled when I first noticed the border guards | 1 and 2 |
| Frightened by Presence of Guards 2 | I was startled by the border guards’ presence | 1 and 2 |
| Frightened by Presence of Guards 3 | The border guards’ presence made me feel stressed | 1 and 2 |
| Frightened by Presence of Guards 4 | The border guards’ presence made me feel tense | 1 and 2 |
| Frightened by Presence of Guards 5 | The border guards’ presence made me feel watched | Only 2 |
| Frightened by Presence of Guards 6 | The border guards’ presence made me feel suspect | Only 2 |
| Cognitive Self-Regulation 1 | During this round I have tried to hide my tension | 1 and 2 |
| Cognitive Self-Regulation 2 | During this round I have tried to hide my nerves | 1 and 2 |
| Cognitive Self-Regulation 3 | During this round I have tried to hide my emotions | 1 and 2 |
| Cognitive Self-Regulation 4 | During this round I have tried not to attract attention | 1 and 2 |
| Cognitive Self-Regulation 5 | During this round I have tried to act as normal as possible | 1 and 2 |
| Awareness Movement Change in Presence of Guards 1 | During this round I have changed my course as soon as I saw the border guards | 1 and 2 |
| Awareness Movement Change in Presence of Guards 2 | During this round I have increased my pace as soon as I saw the border guards | 1 and 2 |
| Suppressed Impulses to Change Movement 1 | I would rather have chosen a different route | 1 and 2 |
| Suppressed Impulses to Change Movement 2 | I would rather have taken a detour to avoid the border guards | 1 and 2 |
| Suppressed Impulses to Change Movement 3 | I would rather have run away from the border guards | 1 and 2 |
| Suppressed Impulses to Change Movement 4 | I would rather have turned around | 1 and 2 |
| Suppressed Impulses to Change Movement 5 | I would rather have hidden myself | 1 and 2 |
| Contemplation of Hostile Intent 1 | I was wondering whether I looked suspicious to the border guards | 1 and 2 |
| Contemplation of Hostile Intent 2 | I was thinking about what I had to hide from the border guards | 1 and 2 |
| Contemplation of Hostile Intent 3 | I was wondering whether I was doing something that I was not allowed to do | 1 and 2 |
| Situational Self Awareness 1 | During this round I was aware of everything in my direct surroundings | 1 and 2 |
| Situational Self Awareness 2 | During this round I was aware of my inner feelings | 1 and 2 |
| Situational Self Awareness 3 | During this round I was aware of the way I presented myself | 1 and 2 |
| Situational Self Awareness 4 | During this round I was aware of how I looked | 1 and 2 |
| Hostile Intent 1 | During this round I felt I was doing something illegal | 1 and 2 |
| Hostile Intent 2 | During this round I felt I had hostile intentions | 1 and 2 |
| Hostile Intent 3 | My role in the experiment made me more tens than usual. | Only 1 |
| Awareness Guard Presence 1 | During this round of the experiment I felt tense because of the presence of the border guards. | Only 1 |
| Awareness Guard Presence 2 | During this round of the experiment I felt nervous because of the presence of the border guards. | Only 1 |
| Awareness Guard Presence 3 | During this round of the experiment I felt watched because of the presence of the border guards. | Only 1 |
| Awareness Guard Presence 4 | During this round of the experiment I felt suspicious because of the presence of the border guards. | Only 1 |
| Other as Target 1 | I had the feeling that the border guards targeted someone else. | Only 1 |
| Other as Target 2 | I had the feeling that the border guards meant someone else. | Only 1 |
| Other as Target 3 | I had the idea that someone else would be stopped. | Only 1 |

Other questions in the questionnaires. The following questions were asked to the participants but not used in the analysis. Some questions were only asked for either on of the two experiments. Since the questionnaire of the first experiment was in Dutch there are also some questions below in Dutch.

Further questions

| Construct | Question | Experiment |
| --- | --- | --- |
| Motivation 1 | I was motivated to obtain a good score in this study | 1 and 2 |
| Motivation 2 | I did the assignment as instructed. | Only 1 |
| Motivation 3 | I was motivated during the execution of the experiment. | Only 1 |
| Strategy Start | What was your strategy in order not to be stopped in the border area? | Only 2 |
| Strategy Finish | What would you do to (further) improve your strategy? | Only 2 |

Leadership question from Experiment 2

| Below you see two rows of squares. In the **top row** (a.), please write down the **GPS tracker numbers** (or **card numbers)** of your fellow team members. In the **bottom row** (b.) please **indicate how much leadership each of your team members have shown**; do so using an index, with 1 indicating the strongest leader, 2 meaning second-strongest leader, etc. Use equal numbers for team members who have shown leadership equally, but please **use index 1 (strongest leader) only once.** | | | | | | |
| --- | --- | --- | --- | --- | --- | --- |
| - 1. Numbers of team members   **[Team1, Team2, …, Team7]** | Team1 | Team2 | Team3 | Team4 | Team5 | Team6 |
|  |  |  |  |  |  |  |
| - 1. Leadership index *(1=strongest, 2=second-strongest, ...)*   **[Leader1, Leader2, … Leader7]** | Leader 1 | Leader2 | Leader3 | Leader4 | Leader5 | Leader6 |
|  |  |  |  |  |  |  |
