# Supplementary material for: “Psyosphere”: A GPS Data-Analysing Tool for the Behavioural Sciences
Source: Front Psychol. 2021 May 13;12:538529. doi: 10.3389/fpsyg.2021.538529 (PMC8155254; doi:10.3389/fpsyg.2021.538529)
Supplement: Supplementary file 3 [file Data_Sheet_3.docx]

## Appendix 3

**Factor Analysis**

We conducted a principal component analysis (PCA) for 30 items of the state questionnaire (see Appendix 1) for each experiment and for each of the first three rounds. This resulted in six PCAs and each PCA used an oblique rotation (oblimin). Afterwards we compared the PCAs by counting how often items were grouped together within a component. We used the resulting model with eight components for a PCA over the data of both experiments and their first three rounds. The Kaiser-Meyer-Olkin (KMO) measure for the PCA verified the sampling adequacy for the analysis. The KMO of .90 is above the minimum of .50 (Kaiser & Rice, 1974). The Bartlett’s test of sphericity, χ² (435) = 8198, p < .001, illustrated that the correlations between the items were large enough for a PCA. The eight components explained 22% of the variance. Seven items had an eigenvalue higher than 1.00 and the eighth component had an eigenvalue of 0.88. As already mentioned, the model that was derived from the first six PCAs indicated eight components and not seven. Additionally, we performed a parallel analysis (Horn, 1965) which had eight components above the simulated and resampled data. Therefore, we decided to retain eight components for the PCA that was conducted for all data combined.

Table 7 shows the factor loadings after rotation in the pattern matrix. The coefficients in the pattern matrix indicate the unique contribution of a component to an item while controlling for other components. Table 8 shows the structure matrix of the PCA. The coefficients in the structure matrix indicate the relationship strength between the item and the component while ignoring other components. The clustering of the items show that the items load on the components as intended. The constructs in order are Alertness to Being Target of Guards, Cognitive Self-Regulation, Situational Self Awareness, Frightened by Presence of Guards, Suppressed Impulses to Change Movement, Contemplation of Hostile Intent, Awareness Movement Change in Presence of Guards and Hostile Intent.

Additionally, we performed a confirmatory factor analysis (CFA) of the state questionnaire. We used maximum likelihood estimation to fit the data of the two experiments. The model fit for Experiment 1 had a CFI of 0.90 and RMSEA of .074, 90% CI (.064, .084). Experiment 2 had a CFI of 0.89 and RMSEA of .071, 90% CI (.064, .077).

To test the invariance of the questionnaire, we made an invariance model comparison of the teams and rounds (Hirschfeld and Von Brachel, 2014). The invariance is tested with four models that increasingly constrain the model variance and are tested in the following sequence: configural invariance (baseline model), weak invariance (constrained factor loadings), strong invariance (constrained factor loadings and intercepts) and strict invariance (constrained factor loading, intercepts and residual variances). The model fits are compared by assessing if the change in CFI is smaller than 0.01 and the Chi-square comparison p-value is larger than 0.05 (Cheung & Rensvold, 2002) if so, it can be assumed that the invariance criteria are met and compare the model fit of the next, more constrained, model.

For the current study, please see Table 9 for the series of model comparison. The invariance model for the teams did not converge and this could have been caused by the complexity of the model and the sample size. The weak invariance model for rounds in Experiment 1 had a significant increase in CFI (Δ CFI = 0.02, p = .020). A closer inspection revealed that the invariance was caused by the first round in Experiment 1, and a reason could be, that participants had many questions about the procedure in the first round of Experiment 1. Therefore, the first round of Experiment 1 was omitted from the invariance analysis. Afterwards, we constrained the factor loadings, intercepts, and residual variances and met the criteria for strict invariance (Δ CFI = .000, p = .374) for Experiment 1. For Experiment 2, we met the criteria for strict invariance without adjustments (Δ CFI = .000, p = .397).

Table 7

*PCA Pattern Matrix for the State Questionnaire*

| Item | Alertness to Being Target of Guards | Cognitive Self-Regulation | Situational Self Awareness | Fright | Suppressed Impulses to Change Movement | Contemplation of Hostile Intent | Awareness Movement Change in Presence of Guards | Hostile Intent |
| --- | --- | --- | --- | --- | --- | --- | --- | --- |
| I thought I had attracted the border guards’ attention | **.89** | -.02 | .02 | -.04 | -.00 | .01 | -.01 | .01 |
| I had the feeling the border guard(s) targeted me | **.89** | .04 | -.02 | -.03 | .03 | .03 | -.01 | -.07 |
| I felt like I was the one being addressed by the border guard(s) | **.87** | -.01 | -.01 | .01 | .04 | .03 | -.02 | .04 |
| I had a feeling that I was going to be stopped | **.79** | .07 | .04 | .05 | .09 | -.02 | -.04 | -.02 |
| I had the idea that the others were paying attention to me | **.70** | -.07 | .04 | .10 | -.13 | -.07 | .19 | .08 |
| During this round I have tried to hide my nerves | .07 | **.88** | -.00 | .09 | -.03 | -.04 | .01 | .03 |
| During this round I have tried to hide my tension | .06 | **.86** | .03 | .03 | -.06 | -.03 | -.02 | .04 |
| During this round I have tried to hide my emotions | .06 | **.84** | -.01 | .03 | .02 | .00 | .03 | .00 |
| During this round I have tried not to attract attention | -.13 | **.79** | -.03 | -.10 | .05 | .11 | .04 | -.06 |
| During this round I have tried to act as normal as possible | -.14 | **.62** | .14 | -.07 | .09 | -.09 | .02 | .08 |
| During this round I was aware of the way I presented myself | .00 | -.02 | **.88** | -.02 | -.04 | .02 | .01 | .05 |
| During this round I was aware of how I looked | -.02 | .07 | **.86** | .01 | -.03 | -.05 | -.03 | -.05 |
| During this round I was aware of my inner feelings | .05 | .04 | **.77** | .06 | .00 | .05 | -.11 | .07 |
| During this round I was aware of everything in my direct surroundings | .02 | -.08 | **.74** | -.02 | .08 | .03 | .10 | -.04 |
| I was startled by the border guards’ presence | .00 | -.04 | .07 | **.81** | .05 | -.14 | .08 | .10 |
| The border guards’ presence made me feel stressed | .06 | .08 | -.02 | **.79** | .06 | .14 | -.11 | .02 |
| I was startled when I first noticed the border guards | .03 | .03 | -.02 | **.79** | .05 | -.17 | .10 | .10 |
| The border guards’ presence made me feel tense | -.01 | .08 | .04 | **.77** | .01 | .27 | -.00 | -.09 |
| I would rather have chosen a different route | .19 | .01 | -.02 | -.13 | **.77** | .02 | -.07 | .06 |
| I would rather have taken a detour to avoid the border guards | .03 | .02 | .06 | .02 | **.77** | .10 | .05 | -.07 |
| I would rather have hidden myself | -.07 | .03 | .02 | .15 | **.72** | .11 | .03 | -.06 |
| I would rather have turned around | .02 | -.00 | -.06 | .16 | **.71** | -.08 | .00 | .14 |
| I would rather have run away from the border guards | .02 | .02 | .05 | .10 | **.52** | -.23 | .24 | .20 |
| I was thinking about what I had to hide from the border guards | .02 | .03 | .12 | -.03 | .07 | **.75** | .14 | .13 |
| I was wondering whether I looked suspicious to the border guards | .05 | .07 | .08 | .22 | .07 | **.61** | .14 | -.12 |
| I was wondering whether I was doing something that I was not allowed to do | .03 | .03 | .02 | -.00 | .07 | **.60** | .01 | **.43** |
| During this round I have increased my pace as soon as I saw the border guards | -.00 | .03 | -.06 | .05 | -.06 | .05 | **.85** | .13 |
| During this round I have changed my course as soon as I saw the border guards | .04 | .06 | .08 | -.03 | .13 | .07 | **.78** | -.16 |
| During this round I felt I was doing something illegal | .02 | .15 | .04 | .06 | .09 | .14 | -.06 | **.74** |
| During this round I felt I had hostile intentions | .02 | .01 | .09 | .11 | .03 | .01 | .12 | **.73** |
| Eigenvalues | 3.70 | 3.52 | 2.92 | 3.08 | 2.96 | 1.79 | 1.74 | 1.77 |
| % of variance | .12 | .12 | .10 | .10 | .10 | .06 | .06 | .06 |
| $\alpha$ (*R*) | .90 | .87 | .84 | .88 | .83 | .78 | (.53) | (.64) |
| *Note.* Component loadings that are higher than or equal to .40 are in bold. Data of all experiments and rounds are analysed together. | | | | | | | | |

Table 8

*PCA Structure Matrix for the State Questionnaire*

| Item | Alertness to Being Target of Guards | Cognitive Self-Regulation | Situational Self Awareness | Frightened by Presence of Guards | Suppressed Impulses to Change Movement | Contemplation of Hostile Intent | Awareness Movement Change in Presence of Guards | Hostile Intent |
| --- | --- | --- | --- | --- | --- | --- | --- | --- |
| I felt like I was the one being addressed by the border guard(s) | **.89** | .13 | .19 | .26 | .38 | .10 | .11 | .20 |
| I thought I had attracted the border guards’ attention | **.88** | .11 | .20 | .20 | .32 | .07 | .09 | .15 |
| I had the feeling the border guard(s) targeted me | **.88** | .15 | .17 | .20 | .35 | .09 | .10 | .09 |
| I had a feeling that I was going to be stopped | **.85** | .22 | .24 | .31 | **.42** | .09 | .12 | .18 |
| I had the idea that the others were paying attention to me | **.71** | .08 | .21 | .30 | .22 | -.00 | .28 | .23 |
| During this round I have tried to hide my nerves | .21 | **.91** | .34 | **.41** | .26 | .22 | .20 | .27 |
| During this round I have tried to hide my tension | .18 | **.87** | .35 | .34 | .20 | .21 | .15 | .25 |
| During this round I have tried to hide my emotions | .20 | **.87** | .32 | .36 | .28 | .25 | .20 | .24 |
| During this round I have tried not to attract attention | -.03 | **.77** | .24 | .17 | .18 | .31 | .15 | .10 |
| During this round I have tried to act as normal as possible | .01 | **.65** | .33 | .20 | .20 | .14 | .15 | .23 |
| During this round I was aware of the way I presented myself | .18 | .30 | **.88** | .17 | .12 | .26 | .20 | .23 |
| During this round I was aware of how I looked | .15 | .34 | **.85** | .16 | .09 | .18 | .15 | .15 |
| During this round I was aware of my inner feelings | .23 | .35 | **.81** | .24 | .18 | .28 | .12 | .27 |
| During this round I was aware of everything in my direct surroundings | .20 | .22 | **.75** | .17 | .21 | .24 | .27 | .16 |
| I was startled by the border guards’ presence | .27 | .29 | .24 | **.88** | .40 | .02 | .36 | **.41** |
| I was startled when I first noticed the border guards | .28 | .31 | .17 | **.87** | .39 | -.02 | .36 | **.41** |
| The border guards’ presence made me feel stressed | .30 | .40 | .22 | **.85** | **.43** | .26 | .20 | .36 |
| The border guards’ presence made me feel tense | .22 | **.42** | .28 | **.81** | .38 | .39 | .29 | .27 |
| I would rather have taken a detour to avoid the border guards | .34 | .27 | .22 | .35 | **.82** | .31 | .29 | .19 |
| I would rather have chosen a different route | **.45** | .19 | .13 | .23 | **.79** | .19 | .14 | .24 |
| I would rather have turned around | .33 | .23 | .10 | **.47** | **.79** | .11 | .25 | .36 |
| I would rather have hidden myself | .25 | .29 | .19 | **.43** | **.78** | .31 | .28 | .21 |
| I would rather have run away from the border guards | .31 | .24 | .20 | **.45** | **.65** | .00 | **.43** | **.40** |
| I was thinking about what I had to hide from the border guards | .16 | .35 | **.41** | .23 | .35 | **.85** | .34 | .32 |
| I was wondering whether I looked suspicious to the border guards | .21 | .37 | .35 | .38 | .36 | **.70** | .34 | .15 |
| I was wondering whether I was doing something that I was not allowed to do | .18 | .33 | .32 | .29 | .35 | **.70** | .22 | **.57** |
| During this round I have increased my pace as soon as I saw the border guards | .11 | .21 | .18 | .34 | .23 | .20 | **.87** | .29 |
| During this round I have changed my course as soon as I saw the border guards | .18 | .24 | .29 | .27 | .35 | .25 | **.82** | .07 |
| During this round I felt I was doing something illegal | .23 | **.41** | .31 | **.43** | .38 | .32 | .18 | **.84** |
| During this round I felt I had hostile intentions | .23 | .29 | .33 | **.46** | .33 | .20 | .33 | **.83** |
| *Note.* Component loadings that are higher than or equal to .40 are in bold. Data of all experiments and rounds are analysed together. | | | | | | | | |

Table 9

*Series of invariance model comparison*

| Model | *χ^2^* (Δ*χ^2^*) | df (Δdf) | *p* (Δ*p*) | CFI (ΔCFI) |
| --- | --- | --- | --- | --- |
| **Experiment 1** |  |  |  |  |
| E1M1 Configural | 1898.3 | 1131 | <.001 | .811 |
| E1M2 Weak invariance | (65.12) | (44) | (.020) | (.005) |
| E1M1b Configural invariance (Round 1 excluded) | 1178.7 | 754 | <.001 | .857 |
| E1M2b Weak invariance (Round 1 excluded) | (26.62) | (22) | .225 | (.001) |
| E1M3b Strong invariance (Round 1 excluded) | (19.89) | (22) | .589 | (.002) |
| E1M4b Strict invariance (Round 1 excluded) | (8.62) | (8) | .374 | (.000) |
| **Experiment 2** |  |  |  |  |
| E2M1 Configural | 1899.7 | 1131 | <.001 | .844 |
| E2M2 Weak invariance | (53.33) | (44) | (.158) | (.002) |
| E2M3 Strong invariance | (54.68) | (44) | (.129) | (.002) |
| E2M4 Strict invariance | (16.82) | (16) | (.397) | (.000) |

**Descriptive Statistics**

Table 10 and Table 11 display preliminary descriptive statistics such as the mean, standard deviation and correlation coefficients for the state constructs and GPS variables per experiment. For example, more participants carried an illegal card (Illegal Card Selection) in Experiment 2 than in Experiment 1 (M1 = 0.41, M2 = 0.88). In Experiment 1 each team had only two illegal cards to distribute, and in Experiment 2 the teams could choose a free ratio of cards and chose on average more illegal cards than in Experiment 1. Also, Hostile Intent was higher in Experiment 2 than in Experiment 1 (M1 = 2.36 M2 = 3.02). To determine the relationship between Illegal Card Selection and Hostile Intent an independent t test was conducted. The test results show that there was a significant difference in Hostile Intent for Experiment 1 when the illegal card was chosen (M1 = 3.07, SD1 = 1.87) than when the legal card was chosen (M1 = 1.88, SD1 = 1.07) with t1 (112) = -5.11, p1 < .001, and Cohen’s d1 = 0.83. Similarly, there was also a significant difference in Hostile Intent for Experiment 2 when the illegal card was chosen (M2 = 3.10, SD2 = 1.64) than when the legal card was chosen (M2 = 2.46, SD2 = 1.31) with t2 (49.04) = -2.60, p2 = .012, and Cohen’s d2 = 0.41. The results of both experiments demonstrate that participants who chose an illegal card reported a higher feeling of hostile intent than participants who chose a legal card, and this relationship was stronger in Experiment 1 than in Experiment 2. A reason for the stronger relationship in Experiment 1 could be that, in Experiment 1, each team had fewer illegal cards to distribute. The illegal cards scored ten points for the team and the legal cards scored one point, therefore, the illegal cards were important to become the team with the highest score. Since the illegal cards were limited in Experiment 1, it was important to carry the illegal cards successfully, that is, without being checked by the guards, to achieve the highest score. By comparison, in Experiment 2, there were unlimited illegal cards which created overall higher self-reported feelings of Hostile Intent but put less stress on the individual that carried an illegal card.

**Differences between experiments.**

The descriptive statistics illustrate some differences between the experiments. For instance, in the first experiment, participants were warned not to run to prevent them from harming themselves. The warning was not given in the second experiment. Consequently, in Experiment 1 the Speed was 4.59 kilometres per hour and 6.01 kilometres per hour in Experiment 2. Additionally, the Speed Variation was 1.43 in Experiment 1 and 4.04 in Experiment 2. It appears that, the warning not to run in Experiment 1 led to a decreased Speed and Speed Variation, compared to Experiment 2.

Other differences between the experiments are the Intra-Team Distance, Route Deviation, and Variation Route Deviation. During Experiment 1 the participants walked closer together than in Experiment 2 (M1 = 9.59, M2 = 12.65). Additionally, the Route Deviation was larger in Experiment 2 than in Experiment 1 (M1 = 7.53, M2 = 11.39), and also, the Variation Route Deviation was larger in Experiment 2 (M1 = 3.15, M2 = 4.77). A likely explanation for the differences, in Intra-Team Distance, Route Deviation, and Variation Route Deviation, could be that the participants in Experiment 2 had to work harder to avoid the guards than in Experiment 1, since there were more guards per participant at the finish area in Experiment 2. Specifically, the ratio between guards and participants was 0.24 in Experiment 1 and 0.54 in Experiment 2. In Experiment 2 were less guards per participant at the finish because in Experiment 1 several teams started at the same time and in Experiment 2 the teams started in turn.

Table 10

*Mean, SD and Correlation of State and GPS Variables for Experiment 1*

|  | Variable |  | *M* | *SD* | 01 | 02 | 03 | 04 | 05 | 06 | 07 | 08 | 09 | 10 | 11 | 12 | 13 | 14 |
| --- | --- | --- | --- | --- | --- | --- | --- | --- | --- | --- | --- | --- | --- | --- | --- | --- | --- | --- |
|  | 01 Illegal Card Selection |  | 0.41 | 0.49 |  | < .001 | .874 | < .001 | .248 | < .001 | .004 | .023 | .612 | .169 | .212 | .468 | .115 | .582 |
|  | 02 Hostile Intent |  | 2.36 | 1.56 | .38 |  | .003 | < .001 | < .001 | < .001 | < .001 | < .001 | < .001 | .468 | .046 | .278 | .631 | .030 |
|  | 03 Alertness to Being Target of Guards |  | 3.71 | 1.72 | -.01 | .21 |  | .126 | .001 | .003 | < .001 | .026 | .875 | .050 | < .001 | .017 | .759 | .561 |
|  | 04 Cognitive Self-Regulation |  | 3.50 | 1.75 | .36 | .41 | .11 |  | < .001 | < .001 | < .001 | < .001 | < .001 | .202 | .877 | .659 | .728 | .944 |
|  | 05 Situational Self Awareness |  | 4.07 | 1.59 | .08 | .36 | .24 | .45 |  | < .001 | < .001 | < .001 | < .001 | .436 | .054 | .289 | .562 | .110 |
|  | 06 Frightened by Presence of Guards |  | 2.73 | 1.42 | .33 | .62 | .23 | .48 | .29 |  | < .001 | < .001 | < .001 | .619 | .390 | .375 | .375 | .441 |
|  | 07 Suppr. Impulses to Change Movement |  | 2.76 | 1.45 | .21 | .65 | .33 | .44 | .33 | .65 |  | < .001 | < .001 | .211 | < .001 | .392 | .151 | .011 |
|  | 08 Contemplation of Hostile Intent |  | 3.40 | 1.50 | .16 | .49 | .16 | .47 | .54 | .46 | .55 |  | < .001 | .561 | .596 | .475 | .977 | .046 |
|  | 09 Awareness Movement Change |  | 3.26 | 1.82 | .04 | .32 | .01 | .30 | .32 | .40 | .37 | .40 |  | < .001 | .260 | .245 | .058 | < .001 |
|  | 10 Speed |  | 4.59 | 0.43 | -.10 | .06 | -.15 | -.10 | .06 | -.04 | -.10 | .04 | .27 |  | < .001 | < .001 | .279 | .001 |
|  | 11 Speed Variation |  | 1.43 | 0.39 | .09 | .15 | .39 | .01 | .15 | .07 | .27 | .04 | -.09 | -.48 |  | .850 | .197 | .490 |
|  | 12 Intra-Team Distance |  | 9.59 | 5.59 | -.05 | .08 | .18 | -.03 | .08 | -.07 | .06 | .05 | .09 | .31 | -.01 |  | .102 | .012 |
|  | 13 Route Deviation |  | 7.47 | 4.06 | -.12 | .04 | .02 | -.03 | .04 | -.07 | .11 | .00 | .14 | .08 | .10 | .12 |  | < .001 |
|  | 14 Variation Route Deviation |  | 3.06 | 1.97 | -.04 | .16 | -.04 | .01 | .12 | .06 | .19 | .15 | .30 | .24 | .05 | .19 | .58 |  |
| *Note*. Upper triangle: *p*-values. Lower triangle: Pearson correlation. | | | | | | | | | | | | | | | | | | |
|  | | | | | | | | | | | | | | | | | | |

Table 11

*Mean, SD and Correlation of State and GPS Variables for Experiment 2*

|  | Variable |  | *M* | *SD* | 01 | 02 | 03 | 04 | 05 | 06 | 07 | 08 | 09 | 10 | 11 | 12 | 13 | 14 |
| --- | --- | --- | --- | --- | --- | --- | --- | --- | --- | --- | --- | --- | --- | --- | --- | --- | --- | --- |
|  | 01 Illegal Card Selection |  | 0.88 | 0.33 |  | .028 | .209 | < .001 | .358 | .010 | .329 | .917 | .368 | .577 | .976 | .261 | .371 | .120 |
|  | 02 Hostile Intent |  | 3.02 | 1.61 | .13 |  | < .001 | < .001 | < .001 | < .001 | < .001 | < .001 | < .001 | .018 | .115 | .437 | .745 | .836 |
|  | 03 Alertness to Being Target of Guards |  | 4.05 | 1.81 | .08 | .30 |  | .001 | < .001 | < .001 | < .001 | < .001 | < .001 | .383 | .089 | .346 | .004 | < .001 |
|  | 04 Cognitive Self-Regulation |  | 3.91 | 1.39 | .24 | .39 | .19 |  | < .001 | < .001 | < .001 | < .001 | < .001 | .474 | .651 | .645 | .354 | .874 |
|  | 05 Situational Self Awareness |  | 4.01 | 1.36 | -.06 | .41 | .28 | .35 |  | < .001 | .005 | < .001 | < .001 | .138 | .602 | .217 | .328 | .965 |
|  | 06 Frightened by Presence of Guards |  | 3.32 | 1.55 | .16 | .48 | .39 | .38 | .28 |  | < .001 | < .001 | < .001 | .442 | .387 | .729 | .647 | .596 |
|  | 07 Suppr. Impulses to Change Movement |  | 2.96 | 1.46 | .06 | .36 | .55 | .22 | .17 | .50 |  | < .001 | < .001 | .161 | .138 | .833 | .084 | .004 |
|  | 08 Contemplation of Hostile Intent |  | 3.32 | 1.54 | .01 | .55 | .31 | .43 | .37 | .41 | .41 |  | < .001 | .227 | .547 | .845 | .927 | .775 |
|  | 09 Awareness Movement Change |  | 3.49 | 1.61 | .05 | .33 | .34 | .23 | .25 | .40 | .43 | .43 |  | .982 | .975 | .522 | .122 | .398 |
|  | 10 Speed |  | 6.01 | 0.84 | .04 | -.16 | -.06 | -.05 | -.10 | -.05 | -.10 | -.08 | .00 |  | < .001 | .535 | .431 | .295 |
|  | 11 Speed Variation |  | 4.04 | 0.96 | .00 | -.11 | .12 | -.03 | -.04 | .06 | .10 | -.04 | .00 | .53 |  | .011 | .877 | .027 |
|  | 12 Intra-Team Distance |  | 12.65 | 4.60 | .08 | .05 | .06 | -.03 | .08 | -.02 | .01 | -.01 | -.04 | .04 | .17 |  | .020 | .030 |
|  | 13 Route Deviation |  | 11.39 | 5.88 | -.06 | .02 | .20 | .06 | .07 | .03 | .12 | -.01 | .11 | .05 | .01 | .16 |  | < .001 |
|  | 14 Variation Route Deviation |  | 4.77 | 2.39 | -.11 | -.01 | .25 | .01 | .00 | -.04 | .20 | -.02 | .06 | .07 | .15 | .15 | .62 |  |

*Note*. Upper triangle: *p*-values. Lower triangle: Pearson correlation.


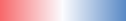


Pearson Correlation

-1.0 1.0


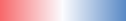


*p*-value

0 1
